# Posttransfusion Sepsis Attributable to Bacterial Contamination in Platelet Collection Set Manufacturing Facility, United States

**DOI:** 10.3201/eid2910.230869

**Published:** 2023-10

**Authors:** Ian Kracalik, Alyssa G. Kent, Carlos H. Villa, Paige Gable, Pallavi Annambhotla, Gillian McAllister, Deborah Yokoe, Charles R. Langelier, Kelly Oakeson, Judith Noble-Wang, Orieji Illoh, Alison Laufer Halpin, Anne F. Eder, Sridhar V. Basavaraju

**Affiliations:** Centers for Disease Control and Prevention, Atlanta, Georgia, USA (I. Kracalik, A.G. Kent, P. Gable, P. Annambhotla, G. McAllister, J. Noble-Wang, A.L. Halpin, S.V. Basavaraju);; Food and Drug Administration, Silver Spring, Maryland, USA (C.H. Villa, O. Illoh, A.F. Eder);; University of California San Francisco School of Medicine, San Francisco, California, USA (D. Yokoe, C.R. Langelier);; Utah Department of Health, Salt Lake City, Utah, USA (K. Oakeson)

**Keywords:** blood safety, posttransfusion sepsis, bacterial contamination, bacteria, Acinetobacter calcoaceticus‒baumannii complex, Staphylococcus saprophyticus, platelets, platelet collection set, blood transfusions, manufacturing facility, United States

## Abstract

During May 2018‒December 2022, we reviewed transfusion-transmitted sepsis cases in the United States attributable to polymicrobial contaminated apheresis platelet components, including *Acinetobacter calcoaceticus‒baumannii* complex or *Staphylococcus saprophyticus* isolated from patients and components. Transfused platelet components underwent bacterial risk control strategies (primary culture, pathogen reduction or primary culture, and secondary rapid test) before transfusion. Environmental samples were collected from a platelet collection set manufacturing facility. Seven sepsis cases from 6 platelet donations from 6 different donors were identified in patients from 6 states; 3 patients died. Cultures identified *Acinetobacter calcoaceticus‒baumannii* complex in 6 patients and 6 transfused platelets, *S. saprophyticus* in 4 patients and 4 transfused platelets. Whole-genome sequencing showed environmental isolates from the manufacturer were closely related genetically to patient and platelet isolates, indicating the manufacturer was the most probable source of recurrent polymicrobial contamination. Clinicians should maintain awareness of possible transfusion-transmitted sepsis even when using bacterial risk control strategies.

In the United States, bacterial contamination of platelet blood components is well documented and largely a consequence of room temperature storage during their 5–7-day shelf life (*1**–**3*). Approximately 2.2 million platelet components are transfused annually in the United States, of which ≈2 million (96%) are collected by using apheresis methods (*4*). Published data from active and passive surveillance indicate bacterial contamination of platelet components (1:2,500‒1:5,000) is more frequent than transfusion-transmitted sepsis (1:10,000‒1:100,000) (*5*). Bacterial contamination of platelet components most commonly occurs during blood collection and typically involves either a single identified species of gram-positive bacteria associated with normal skin microflora or, less commonly, gram-negative bacteria from asymptomatic donor bacteremia. However, multiple episodes of polymicrobial contamination with identical bacterial species in platelet components across different states is exceedingly rare, suggesting a possible common source of contamination. One platelet donation can yield up to 3 platelet components, and each component might cause septic reactions in different patients (*2*,*6*,*7*). Signs and symptoms of transfusion-transmitted sepsis typically occur within minutes to hours after transfusion and include fever, chills, and hypotension; such reactions might be severe or fatal, although many recipients of bacterially contaminated platelets remain asymptomatic (*3*).

Strategies to mitigate sepsis risk caused by bacterial contamination of platelets include bacterial cultures incubated before release for transfusion, secondary rapid testing after bacterial culture with a bacterial detection device, and pathogen reduction after platelet collection (*8*). In the United States, a pathogen-reduction device for platelets that uses synthetic psoralen and ultraviolet light to inactivate microorganisms was approved by the US Food and Drug Administration (FDA) in 2014 and adopted voluntarily by some blood establishments. In response to ongoing reports of transfusion-transmitted sepsis, FDA established regulations and recommendations in guidance during September 2019 to implement certain bacterial risk control strategies for platelets collected before October 1, 2021, including pathogen reduction, bacterial culture methods, and secondary rapid testing (*8*).

We report on a multistate investigation in the United States of transfusion-transmitted sepsis involving 2 uncommon contaminants of platelet blood components, *Acinetobacter calcoaceticus-baumannii* complex (ACBC) or *Staphylococcus saprophyticus*, and other species (e.g., *Leclercia adecarboxylata*) seen in combination among recipients of apheresis platelet components (not pathogen reduced) and pathogen-reduced apheresis platelet components. We describe the epidemiologic and laboratory efforts to identify the source of platelet contamination.

## Methods

### Overview of the Investigation

**Figure 1:**
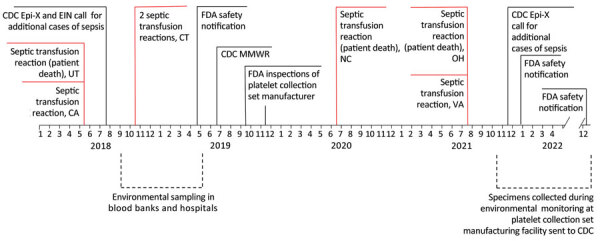
Investigation timeline of transfusion-transmitted sepsis cases and key events for bacterial contamination in platelet collection set manufacturing facilities, United States, 2018–2022. CDC, Centers for Disease Control and Prevention; EIN, Emerging Infections Network; Epi-X, Epidemic Information Exchange; FDA, Food and Drug Administration; MMWR, report published in Morbidity and Mortality Weekly Report (*9*).

During May–October 2018, the Centers for Disease Control and Prevention (CDC) and FDA received 4 reports of transfusion-transmitted sepsis attributable to platelet components collected by 1 blood establishment from 3 donors at separate collection facilities in 3 states. Implicated platelet components underwent bacterial risk mitigation strategies before transfusion, including primary bacterial culture, pathogen reduction or a combination of primary culture, and secondary rapid testing (Table). Preliminary whole-genome sequencing (WGS) showed that respective species isolates were closely related genetically, suggesting a common source of contamination. CDC issued 2 calls for cases through the Epidemic Information Exchange (Figure 1).

**Table:** Characteristic of confirmed and probable transfusion-transmitted sepsis associated with bacterially contaminated platelet products for 7 patients, United States, 2018–2021*

Characteristic	Patient A	Patient B	Patient C	Patient D	Patient E	Patient F	Patient G
Sex	M	M	M	M	M	F	F
State	CA	UT	CT	CT	NC	OH	VA
Year	2018	2018	2018	2018	2020	2021	2021
Posttransfusion clinical status	Alive	Deceased	Alive	Alive	Deceased	Deceased	Alive
Platelet collection system	Amicus	Amicus	Amicus	Amicus	Amicus	Amicus	Amicus
Platelet storage media	PA	PAS	PAS	PAS	PAS	PAS	PAS
Bacterial control strategy	PR	PC	PC, RT	PC, RT	PR	PR	PR
Platelet age, d	5	5	4	4	5	5	4
Platelet co-component transfused	No	No	Yes	Yes	Yes	Yes	Yes
Posttransfusion cultures							
Patient blood							
ACBC	Yes	Yes	Yes	Yes	Yes	Yes	No
* Staphylococcus saprophyticus*	No	No	Yes	Yes	Yes	Yes	No
* Leclercia adecarboxylata*	No	No	No	No	Yes	No	No
*Enterobacter* spp.	No	No	No	No	No	No	Yes
Transfused platelet product							
ACBC	Yes	Yes	Yes	Yes	Yes	Yes	No
* S. saprophyticus*	Yes	No	Yes	Yes	Yes	Yes	Yes
* L. adecarboxylata*	No	No	No	No	Yes	Yes	Yes
*Enterobacter* spp.	No	No	No	No	No	No	Yes
*Bacillus* spp.	No	No	No	No	No	Yes	No
Signs and symptoms							
Fever	Yes	Yes	Yes	Yes	Yes	No	Yes
Hypotension	Yes	Yes	Yes	Yes	Yes	Yes	Yes
Chills	Yes	Yes	Yes	Yes	No	No	Yes
Tachycardia or bradycardia	Yes	Yes	No	No	Yes	Yes	Yes

*Platelets were stored in a combination of platelet additive solution and plasma. Patients C and D received platelet components from the same platelet collection. Amicus platelet collection sets and associated solutions (saline and anticoagulant) are manufactured by Fenwal International, Inc., a Fresenius Kabi Company (https://www.fresenius-kabi.com). ACBC, *Acinetobacter calcoaceticus‒baumannii* complex; PAS, platelet additive solution; PC, primary culture; PR, pathogen reduction; RT, secondary rapid test.

On April 16, 2019, FDA issued a safety communication, updated on December 2, 2021 and December 22, 2022, encouraging blood establishments and healthcare facilities to report platelets contaminated with ACBC or *S*. *saprophyticus* by submitting a MedWatch report or by directly contacting FDA Center for Biologics Evaluation and Research (*10**,**11*). As the investigation progressed, additional reports identified *L. adecarboxylata* as a platelet contaminant in combination with ACBC or *S*. *saprophyticus*. Beginning in May 2021, isolates identified during routine environmental sampling by a platelet collection set manufacturer were sent to CDC for testing.

This activity was determined to meet the requirements of public health surveillance as defined in Title 45 of the Code of Federal Regulations, part 46.102(l) (*2*). No institutional review board approval was needed.

### Identification of Transfusion-Transmitted Sepsis Cases

We identified cases of transfusion-transmitted sepsis through mandatory reporting of transfusion-related deaths to the FDA under 21 CFR 606.170(b) or voluntary reports to the CDC or FDA by US blood establishments, health departments, or healthcare facilities. We reviewed reports of transfusion-transmitted sepsis for case definition and imputability criteria contained within the National Healthcare Safety Network Hemovigilance Module protocol (Appendix). We included cases if identical bacterial species were isolated from a transfused patient and a transfused platelet component, and an implicated strain (ACBC or *S*. *saprophyticus*) was isolated from either a transfused patient or transfused platelet component.

### Contaminated Platelets

Blood establishments or healthcare facilities voluntarily reported, to the FDA or CDC, platelets contaminated with ACBC or *S*. *saprophyticus* identified by primary bacterial culture screening before distribution; contaminated units were not released for transfusion. As required under 21 CFR 606.171. Information collected on patients and contaminated platelet components included the blood collection establishment, date and location of donation, collection method and platform, collection kit manufacturer, use of additive solution, and bacterial risk control strategy.

### Environmental Sampling

We reviewed focused environmental surface sampling (Appendix) conducted by CDC and local and state health departments in blood establishments and healthcare facilities in 5 US states (California, Connecticut, Massachusetts, North Carolina, and Utah) from which platelet components were collected, or in hospitals in which cases of transfusion-transmitted sepsis were reported. We used epidemiologic data collected during the investigation to identify potential reservoirs, and sampling locations, such as equipment used to store platelet components.

As part of routine environmental monitoring procedures, the manufacturer (Fenwal International, Inc., a Fresenius Kabi Company, https://www.fresenius-kabi.com) of Amicus platelet collection sets and associated solutions (saline and anticoagulant) collected environmental isolates in 2 of their platelet collection set manufacturing facilities located in Puerto Rico and the Dominican Republic. A subset of the manufacturer’s environmental isolates were sent to CDC for species verification and WGS.

### Microbiologic Investigation

Microbiologic characterization included isolates from patients, implicated transfused platelet components, contaminated platelet products identified before transfusion, and environmental sampling. We performed species identification by using matrix-assisted laser desorption ionization–time-of-flight mass spectrometry (Appendix). Polymicrobial contamination was defined as identification of >1 species from a platelet component or patient.

### WGS and Analysis

CDC or state health departments performed WGS. At CDC, we used Maxwell 16 MDx System (Promega, https://www.promega.com) to extract genomic DNA. We created DNA libraries by using the NuGEN Ovation Ultralow V2 System (Agilent, https://www.agilent.com). We performed DNA sequencing on the Illumina MiSeq Sequencing platform (https://www.illumina.com). We performed quality control, preliminary analyses, and high-quality single-nucleotide variant (hqSNV) analysis by using the QuAISAR-H Pipeline (https://github.com/DHQP/QuAISAR_singularity) and SNVPhyl (https://snvphyl.readthedocs.io) (Appendix) (*12*).

We queried the National Center for Biotechnology Information (NCBI) RefSeq database (https://www.ncbi.nlm.nih.gov/refseq), which yielded 7,921 ACBC and 160 *S. saprophyticus* genomes. We compared NCBI data with isolates implicated in cases of sepsis by using a maximum-likelihood phylogenetic method based on core genes (Appendix).

One ACBC isolate cultured from the environment in a platelet collection set manufacturing facility underwent multilocus sequence typing at an outside laboratory. Results were sent to CDC for additional analysis, but the isolate was not available for WGS.

## Results

### Transfusion-Transmitted Bacterial Sepsis Cases

During May 2018–November 2022, a total of 7 cases of platelet transfusion-transmitted sepsis were identified in patients from 6 states (California, Utah, Connecticut, North Carolina, Ohio, and Virginia) (Table). Some of those cases have been previously reported but are included here for completeness and context in the larger investigation (*9**,**13**‒**15*). Of the cases, 3 were identified through reports to FDA of transfusion-related fatalities under 21CFR 606.170(b); others were voluntarily reported by state health departments or blood establishments. Other than receipt of platelet transfusion, no commonalities were observed among persons who died.

Blood establishments collected platelets implicated in transfusion-transmitted sepsis by using apheresis on the Amicus platform (Fenwal International, Inc.) and suspended these platelets in 65% platelet additive solution (InterSol Solution/Platelet Additive Solution 3 [PAS-C]; Fenwal International, Inc.) and 35% plasma. Platelets were collected from 6 different donors in 6 states, all by 1 blood establishment. Two of the implicated platelet units were from the same collection procedure. FDA traced lot numbers of all materials used in the collections, including collection sets, anticoagulant solutions, saline, additive solutions, and sampling devices, to identify potential common elements. Platelet collection sets were from a single manufacturer consisting of multiple lot numbers of collection sets, saline, anti-coagulant solution, and PAS-C manufactured during 2018‒2020. Blood establishments performed various measures (e.g., terminal cleaning) to reduce the risk for bacterial contamination of all platelet components.

Of the 7 platelet components implicated in sepsis, 4 (57%) were pathogen reduced and 3 (43%) underwent primary bacterial culture at least 24 hours after collection and were negative; of those 3 components, 2 (67%) also underwent secondary rapid testing at day 4 and were negative (Table). Polymicrobial contamination was identified in 6/7 (86%) cases; in 1 case, only ACBC was isolated. Posttransfusion blood cultures identified ACBC in 6/7 (86%) patients, *S. saprophyticus* in 4/7 (57%) patients, *L. adecarboxylata* in 1/7 (14%) patients, and *Enterobacter* spp. in 1/7 (14%) patients. Cultures from implicated platelet components identified ACBC in 6/7 (86%) cultures, *S. saprophyticus* in 6/7 (86%) cultures, *L. adecarboxylata* in 3/7 (43%) cultures, *Enterobacter* spp. in 1/7 (14%) cultures, and *Bacillus* spp. in 1/7 (14%) cultures (Table).

Disposition of co-components from the 7 cases of transfusion-transmitted sepsis included 3/7 (43%) platelet co-components transfused into 3 other patients without incident, as reported by blood establishments or transfusion services; 2/7 (14%) sequestered co-components that were culture negative; and 2/7 (14%) co-components that caused septic reactions were part of this investigation (patients C and D) (Table). Blood establishments or transfusion services did not report additional details of their investigation into the co-component transfusions. In patients in whom septic reactions were observed, onset of symptoms ranged from 30 minutes to 3 hours after start of transfusion and most frequently included hypotension (7/7, 100%) and fever (6/7, 86%).

### Bacterially Contaminated Platelet Components

**Figure 2:**
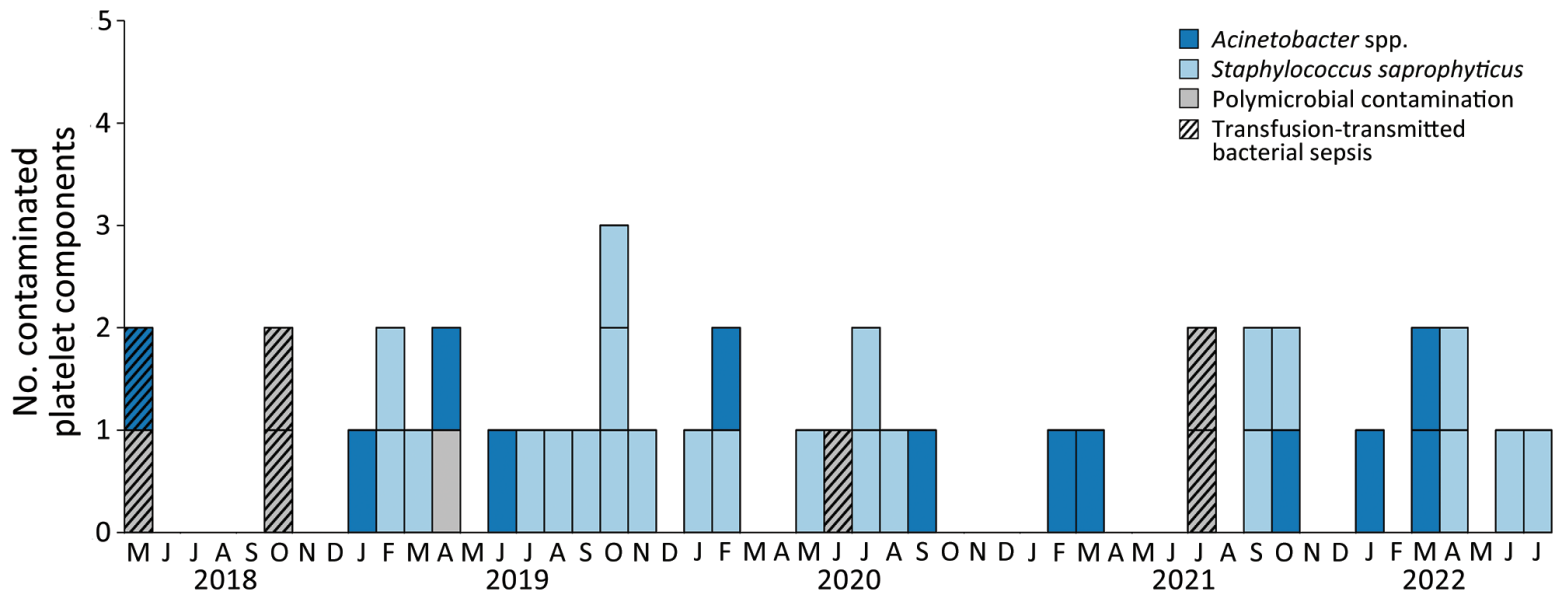
Platelet components contaminated with *Acinetobacter* spp. or *Staphylococcus saprophyticus* identified from cases of transfusion-transmitted bacterial sepsis or routine bacterial testing before transfusion, United States, 2018–2022.

During February 2019–November 2022, primary bacterial culture identified, before transfusion, 38 isolates from 35 nonpathogen-reduced apheresis platelet components contaminated with *Acinetobacter* spp. or *S. saprophyticus* collected in 12 states (Arizona, California, Connecticut, Massachusetts, Maryland, Missouri, New York, North Carolina, Ohio, Pennsylvania, Texas, and Wisconsin). Of those samples, 21/35 (60%) were contaminated with *S. saprophyticus*, 11/35 (31%) with *Acinetobacter* spp., 2/35 (6%) each with 2 different strains of *S. saprophyticus*, and 1/35 (3%) with both *S. saprophyticus* and *Acinetobacter* spp. (Figure 2).

Contaminated platelet components had been suspended in 100% plasma (20/35, 57%) or 65% PAS-C/35% plasma (15/35, 43%). A total of 29/35 (83%) contaminated platelet components interdicted before transfusion were collected by using the Amicus Platform by 1 blood establishment; 6/35 (17%) were collected on the Trima Accel Platform (Terumo BCT, https://www.terumobct.com) by a second blood establishment. One additional platelet component collected on the Trima Platform was transfused into a patient, and *A. radioresistens* was cultured from the remaining platelet component, which underwent WGS. However, that case did not meet the transfusion-transmitted sepsis case definition and imputability criteria (Appendix), and the isolate did not cluster with other strains implicated in cases of sepsis. No commonalities (e.g., solutions or storage containers) between contaminated components from different blood establishments or collection set manufacturers were identified.

### Environmental Sampling

A total of 90 environmental samples were collected from blood establishment and healthcare facilities in 5 states (California, Connecticut, Massachusetts, North Carolina, and Utah) during May, June, and November 2018; February and May 2019; and July 2020. Of the 90 samples cultured, 29 (32%) yielded 34 implicated strain isolates. Recovery of isolates was primarily associated with samples taken from equipment used to store (e.g., platelet agitators) and transport platelet components (e.g., quality control cart) (Appendix Table 1). Of the 34 isolates, 19 (56%) were ACBC, 11 (32%) were *S. saprophyticus*, and 4 (12%) were *L. adecarboxylata*.

Because cases of transfusion-transmitted sepsis all involved collection sets and solutions (i.e., anticoagulant solution, saline, PAS-C) from the same manufacturer, FDA inspected the manufacturing facilities in Puerto Rico and the Dominican Republic to assess the risk for a common source of contamination. As part of those activities, during October 2021–October 2022, additional culture and sequencing was performed on 74 environmental samples collected at the 2 manufacturing facilities (Appendix Table 2), yielding 84 isolates: 35 *Acinetobacter* spp. and 49 *S. saprophyticus*. FDA inspections of the manufacturing facilities identified deficiencies related to environmental controls and the assurance of platelet collection set sterility (*16*).

### WGS and Analysis

**Figure 3:**
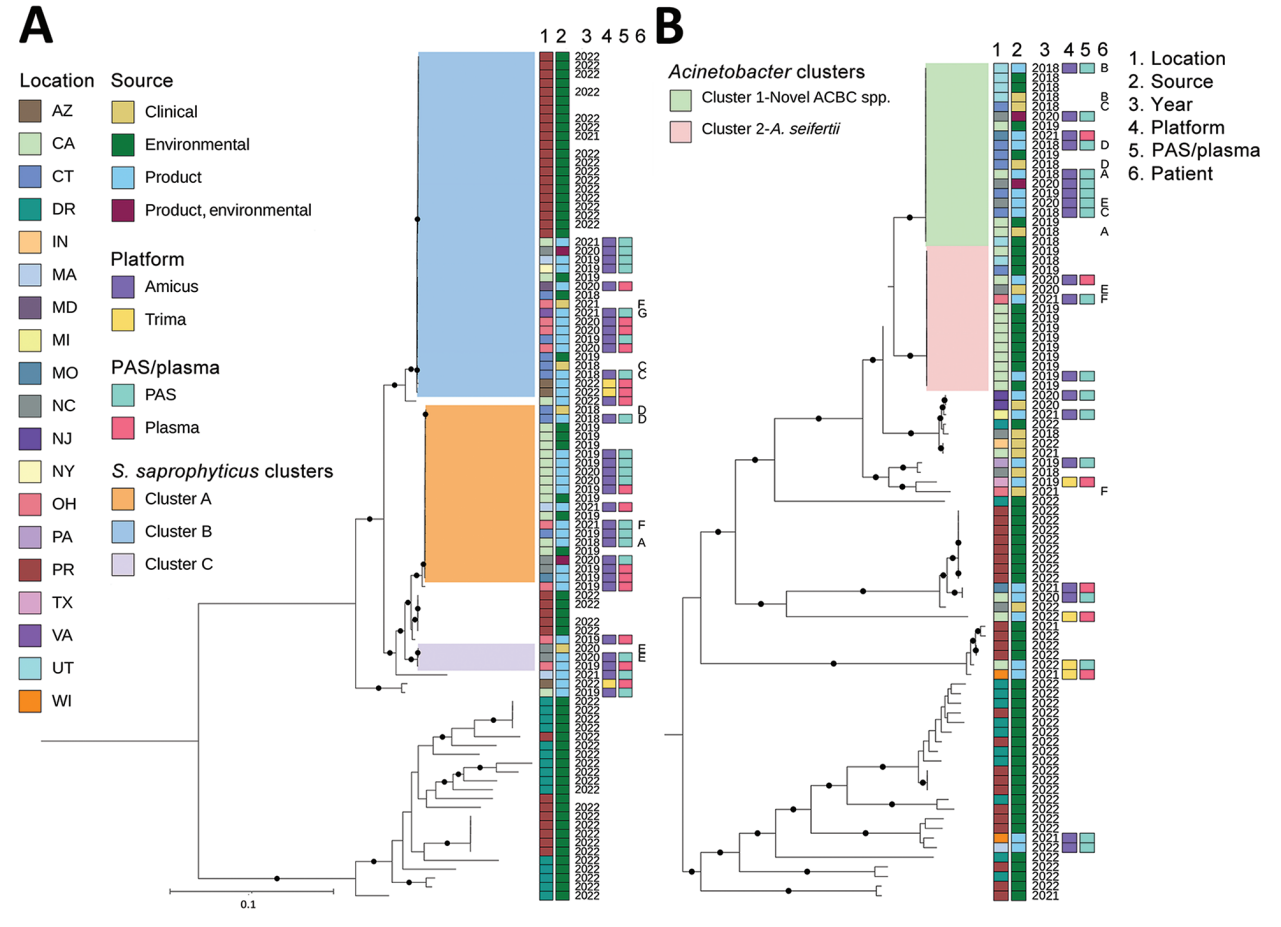
Whole-genome sequencing of *Staphylococcus saprophyticus* (A) and ACBC (B) isolates implicated in the bacterial contamination of platelet blood products, United States, 2018–2022. Maximum-likelihood phylogenies based on core genes were generated by using Roary (https://github.com/sanger-pathogens/Roary) and RaxML (https://cme.h-its.org); phylogenetic trees were midpoint rooted. Clusters were identified based on SNVPhyl (https://snvphyl.readthedocs.io) and highlighted if they included isolates linked to a sepsis transfusion case. *Acinetobacter* spp. isolates not falling in the ACBC were also included. Black circles on branches indicate 100% support for the branch of 100 bootstraps. US states are identified by 2-letter postal codes. Scale bars indicate nucleotide substitutions per site. ACBC, *Acinetobacter calcoaceticus–baumannii* complex; DR, Dominican Republic; PAS, platelet additive solution; PR, Puerto Rico.

A total of 191 isolates obtained over 4 years underwent WGS: 118 environmental isolates from healthcare facilities, blood establishments and 2 platelet collection set manufacturing facilities; 56 from contaminated platelet components; and 17 from posttransfusion patient blood (Figure 3). Sequencing and analysis showed that respective isolates of ACBC, *S. saprophyticus*, and *L. adecarboxylata* from different sources were closely related genetically and formed several closely related, respective outbreak clusters. Isolates from posttransfusion patient blood, platelet components, and a platelet collection set manufacturing facility formed 3 distinct *S. saprophyticus* outbreak clusters: cluster A (20 isolates, differing by 1–38 pairwise hqSNVs, across 97.9% of the reference isolate core genome), cluster B (39 isolates, 0–48 hqSNVs, 98.9% core genome) and cluster C (3 isolates, 0–32 hqSNVs, 99.0% core genome).

Isolates from patient blood, platelet components, and the hospital/blood establishment environment were distributed across 2 distinct ACBC outbreak clusters: cluster 1, a potentially novel species (closest average nucleotide identity to *A. seifertii* at 90.8%) within ACBC (19 isolates, varied by 0–79 hqSNVs, 94.9% core genome) and cluster 2, *A. seifertii* (15 isolates, 5–129 hqSNVs, 95.2% core genome). For the *L. adecarboxylata* isolates, sequencing and analysis showed 1 outbreak cluster (5 isolates, 1–32 hqSNVs, 97.2% core genome).

Of the 7 cases of transfusion-transmitted sepsis, all were collected on the Amicus platform and had at least 1 implicated bacterial isolate in the investigation cluster. Of the primary culture positive units (not clinical cases), most units collected on the Amicus platform were in the investigation clusters (20/29 components; 69%), but only 2/6 units (33%) collected on the Trima platform were in the investigation clusters. Furthermore, these 2 Trima isolates were collected in the same location (Appendix Figure).

**Figure 4:**
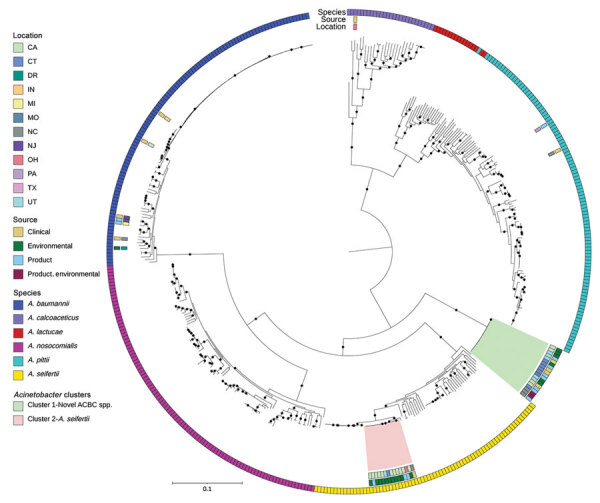
Public ACBC shown with study isolates from investigation of bacterial contamination of platelet blood products, United States, 2018–2022. Shown is a RaxML (https://cme.h-its.org)‒generated phylogeny based on core genes of genomes from ACBC isolates from this study compared with all *A. calcoaceticus*, *A. lactucae*, and *A. seifertii* and a subsampled set of *A. baumannii, A. nosocomialis*, and *A. pittii* genomes from the National Center for Biotechnology Information RefSeq (https://www.ncbi.nlm.nih.gov/refseq) database, along with all *Staphylococcus saprophyticus* from the RefSeq database. Isolate location, isolate source, and species from National Center for Biotechnology Information database along with all *S. saprophyticus* isolates or by average nucleotide identity were layered onto the phylogeny. Pink and green indicate the 2 clusters from Figure 3, panel B. Black circles on branches indicate 100% support for the branch of 100 bootstraps. US states are identified by 2-letter postal codes. Scale bar indicates nucleotide substitutions per site. ACBC, *Acinetobacter calcoaceticus-baumannii*; DR, Dominican Republic.

**Figure 5:**
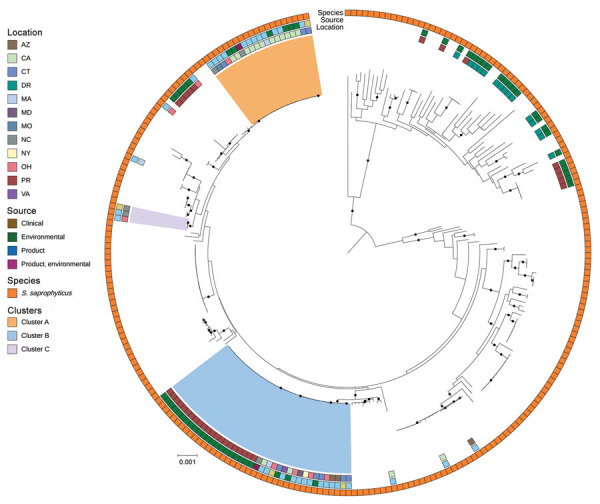
Public *Staphylococcus saprophyticus* genomes and study isolates from investigation of bacterial contamination of platelet blood products, United States, 2018–2022. Shown is a RaxML (https://cme.h-its.org)‒generated phylogeny based on 1,808 core genes of all *S. saprophyticus* isolates from this study and all *S.saprophyticus* genomes from the National Center for Biotechnology Information RefSeq (https://www.ncbi.nlm.nih.gov/refseq) database. Isolate location, isolate source, and species from National Center for Biotechnology Information or by average nucleotide identity were layered onto the phylogeny. Light orange, blue, and purple indicate the 3 clusters from Figure 3, panel A. Black circles on branches indicate 100% support for the branch of 100 bootstraps. US states are identified by 2-letter postal codes. Scale bar indicates nucleotide substitutions per site. DR, Dominican Republic; PR, Puerto Rico.

One ACBC isolate obtained in July 2022 from a platelet collection set manufacturer was the same potentially novel ACBC species in outbreak cluster 1 and was closely related by multilocus sequence typing, but was not available for WGS and analysis at CDC. In the NCBI, ACBC isolates from cluster 1, which included this potentially novel species, grouped distinctly from all other ACBC with available data (Figure 4). All outbreak clusters in ACBC and *S. saprophyticus* clustered apart from publicly available NCBI genomes (Figure 4, Figure 5).

## Discussion

In this investigation, we found that a platelet collection set manufacturer was the most probable source of multiple episodes of polymicrobial contamination of platelet components. Previous, large, multiyear studies of platelet collections and transfusions did not show polymicrobial contamination (*7*,*17*,*18*). During the investigation, primary culture of some platelet collections by blood establishments, before transfusion, identified bacterial contamination with implicated strains, thus averting possible septic reactions. However, despite the use of bacterial risk control strategies, including primary culture, secondary rapid testing, or pathogen reduction, 7 cases of transfusion-transmitted sepsis were attributed to bacterial contamination of platelet components and resulted in 3 deaths. The manufacturer of the secondary rapid test has since updated the device to improve detection of ACBC (*19*). Using pathogen reduction or other detection-based bacterial risk control strategies cannot eliminate the risk for transfusion-transmitted sepsis; however, the risk is expected to be reduced by those measures and remains low (*2*,*5*,*20*,*21*). Of the ≈2.1 million apheresis platelet units transfused in 2021, ≈843,000 were pathogen reduced, and ≈1.2 million underwent various bacterial testing methods (*4*), suggesting the cases described in our study were rare events.

FDA continues to conduct inspections of the manufacturer to ensure control of the manufacturing process and maintain sterility of platelet collection sets and solutions. FDA has communicated that strategies to ensure the bacterial safety of platelet components recommended in FDA guidance remain acceptable at this time (*8**,**10**,**11*). Because some platelet donations in this investigation were tested before the implementation date of the December 2020 guidance on bacterial risk control strategies, bacterial culture methods consistent with the guidance might not have been implemented by blood establishments.

Identifying the source of platelet contamination was challenging. We initially hypothesized the contamination was an isolated event resulting from donor infection or colonization. Only 1 previously reported cluster of transfusion-transmitted sepsis has been attributed to bacterial contamination from a common source and implicated blood collection sets (*22*). Our investigation then focused on environmental contamination in hospitals and blood establishments; however, identification of genetically related implicated strains, temporally and geographically dispersed, shifted our attention to a common source contamination at the level of materials used in manufacturing of platelet components. Another challenge was the complex chain of blood donation and product manufacturing, including collection kit (e.g., platelet collection bags and additive solutions) manufacturers and kit assembly facilities across 3 locations. Traceback efforts initiated in 2018 did not identify a putative contamination source. Not until 2021 were environmental isolates identified at a platelet collection set manufacturing facility and subsequently sequenced in 2022 to identify isolates situated within the *S. saprophyticus* outbreak-related clusters.

Before this investigation, there were no confirmed reports of sepsis attributable to bacterial contamination of pathogen-reduced platelets (*23*). Published studies hypothesized that cases of sepsis in this outbreak were caused by defective platelet containers, resulting in environmental contamination after pathogen reduction (*14*,*15*,*24*). However, several lines of evidence from this investigation suggest a common source of contamination occurring before pathogen reduction. First, WGS showed geographically and temporally dispersed isolates from varying sources, including patients, platelet components, and a platelet collection set manufacturer, were closely related genetically. Moreover, a possible novel ACBC species, not previously contained within public repositories, was identified in patients and the environment, irrespective of time and place (*13*,*25*). Second, implicated strains were identified before transfusion and storage of platelets that had not been pathogen reduced; those platelet components did not have the same final container as pathogen-reduced platelets, for which defects have been hypothesized as a contamination source (*15*,*24*). Contaminated platelet components were suspended in either 100% plasma or 65% PAS-C/35% plasma, suggesting other commonalities (e.g., saline or anticoagulant solutions) were sources of contamination, which was supported by environmental sampling and inspections identifying manufacturing deficiencies related to the assurance of sterility.

ACBC and *S. saprophyticus* are uncommon platelet contaminants and together have not previously been reported as polymicrobial contaminants implicated in transfusion-transmitted sepsis (*2*,*3*,*21*). ACBC are widely distributed gram-negative organisms found in moist environments that can persist on surfaces and adhere to plastics (*26*). Although coagulase-negative *Staphylococcus* spp. are more frequent platelet contaminants, those bacteria are often identified as *S. epidermidis* and not *S. saprophyticus* (*6*,*18*). *S. saprophyticus* commonly colonizes the human urogenital tract and is a leading cause of urinary tract infections but is not typically implicated in transfusion-transmitted sepsis (*27*).

Both ACBC and *S. saprophyticus* have been shown to contaminate the environment and can form biofilms (*27**–**29*), although synergistic growth enhancement between ACBC and *S. saprophyticus* has not been observed (*30*). Effective pathogen reduction was demonstrated in platelet components inoculated with implicated strains of ACBC and *S. saprophyticus,* but that finding might not reflect real-world conditions in clinical practice during this investigation (*14*,*15*). However, 1 study detected residual viable bacteria after 7 days of storage in pathogen-reduced platelets experimentally inoculated with *L. adecarboxylata*, which was attributed to a high inoculum (*26*). Certain spore-forming bacteria and nonenveloped viruses have also demonstrated resistance to pathogen reduction (*23*), and biofilm-forming bacteria might evade detection by bacterial culture or pathogen reduction (*31*).

One limitation of our findings is that, although efforts were made to identify cases or contaminated products, some might have gone unrecognized. Some patients lack clinical symptoms of transfusion-transmitted sepsis caused by varying inoculum size, asymptomatic infection, or misdiagnosis (*5*,*32*). Highlighting this variability is that, for some platelet collections associated with fatal sepsis in this investigation, platelet co-components from the same donation were transfused into 3 additional patients without incident. Pathogen-reduced platelets do not routinely undergo primary culture for bacteria before transfusion; consequently, the bacterial load is unknown. However, pathogen reduction of some platelet components during this investigation probably prevented some cases of transfusion-transmitted sepsis. The mechanisms of pathogen reduction evasion are unclear, and the effectiveness of pathogen reduction against biofilms is not completely understood.

Although most contaminated platelet components in this outbreak were collected by 1 blood establishment on the Amicus platform, 6 isolates were interdicted from components collected by a second blood establishment on the Trima platform, and 2 of the 6 were closely related genetically to the *S. saprophyticus* cluster. However, those 2 isolates showed evidence of a common plasmid not identified in any other isolates in the cluster (data not shown). Furthermore, no epidemiologic links (e.g., shared materials or devices) were identified between this second establishment and the first establishment’s outbreak-related transfusion transmitted sepsis cases or platelet components, despite association with the common *S. saprophyticus* strain. In contrast, strains associated with polymicrobial contamination were common among clinical cases of sepsis and the implicated manufacturing facility.

CDC and FDA continue to collaborate to ensure the safety of the blood supply. CDC has proposed additional studies to elucidate the effects of biofilm development on platelet bacterial risk control strategies (*33*). FDA and CDC will continue to monitor reports of bacterial contamination of platelets, and FDA will issue additional communications and take appropriate regulatory action as necessary. Despite these events, blood transfusion remains safer than ever because of bacterial mitigation strategies. Although such cases are uncommon, clinicians should maintain awareness of possible transfusion-transmitted sepsis even when using bacterial risk control strategies. Prompt recognition and reporting will help ensure identification of potential sources of contamination so that appropriate corrective actions can be taken.

Appendix Additional information on posttransfusion sepsis attributable to bacterial contamination in platelet collection set manufacturing facility, United States.
