# Supplementary material for: Posttransfusion Sepsis Attributable to Bacterial Contamination in Platelet Collection Set Manufacturing Facility, United States
Source: Emerg Infect Dis. 2023 Oct;29(10):1979–89. doi: 10.3201/eid2910.230869 (PMC10521617; doi:10.3201/eid2910.230869)
Supplement: Appendix — Additional information on posttransfusion sepsis attributable to bacterial contamination in platelet collection set manufacturing facility, United States. [file 23-0869-Techapp-s1.pdf]

Article DOI: https://doi.org/10.3201/eid2910.230869

EID cannot ensure accessibility for Supplemental Materials supplied by authors. Readers who have difficulty accessing supplementary content should contact the authors for assistance.

Posttransfusion Sepsis Attributable to Bacterial Contamination in Platelet Collection Set Manufacturing Facility, United States

Appendix

Identification of Transfusion-Transmitted Sepsis Cases

Cases of transfusion-transmitted sepsis were reviewed for case definition and imputability criteria using the National Healthcare Safety Network Hemovigilance Module. Cases were included that met definite case definition and definite imputability criteria and an implicated strain (ACBC or *S. saprophyticus*) was isolated from either a transfused patient or transfused platelet component. A definite case was defined as laboratory evidence of a pathogen in a recipient of a platelet component. Definite imputability criteria was defined as one or more of the following: evidence of the pathogen in the transfused platelet component; evidence of the pathogen in the donor at the time of donation; evidence of the pathogen in an additional platelet component from the same donation; evidence of the pathogen in an additional recipient of a platelet component from the same donation; and no other potential exposures to the pathogen could be identified in the recipient; and either evidence that the recipient was not infected with the pathogen before transfusion; or evidence that the identified pathogen strains are related by molecular or extended phenotypic comparison testing with statistical confidence ($p<0.05$).

Bacterial Species Identification and Whole-Genome Sequencing

Species-level identification was performed by using matrix-assisted laser desorption ionization–time of flight (MALDI-TOF) (MALDI Biotyper, Bruker Daltonics, Billerica, MA) and is considered confident for scores ≥2.0. Genomic DNA were extracted by using the Maxwell 16 MDx system (Promega, Madison, WI) and DNA was sheared by using the Covaris ME220 (Woburn, MA) to ≈600 bp. Sequencing using the Illumina MiSeq (San Diego, CA) MiSeq

reagent kit v2 yielded 250 bp paired-end reads or Illumina NextSeq 550 (San Diego, CA) which yielded 150 bp paired-end reads. Sequences were deposited in the NCBI Sequence Read Archive (SRA) under bioprojects PRJNA288601 and PRJNA591020 with BioSample accessions from SAMN36357091 to SAMN36357280 and SAMN36619280. Raw sequencing reads were processed through CDC's in-house QuAISAR-H pipeline; all publicly available tools and versions can be found on the QuAISAR-H repository (https://github.com/DHQP/QuAISAR_singularity). Specifically, sequences were processed with BBDuk v38.90 and Fastp v0.20.1 to remove adapters and PhiX, and quality trim sequences (*1*,*2*). Samples were assembled de novo using SPAdes v3.15.0 (*3*). After assembly, scaffolds less than 500 bp were removed. Taxonomic identification used pyANI v.0.2.10 to find the highest Average Nucleotide Identity (ANI) against NCBI's RefSeq database (*4*). Maximum likelihood phylogenetic trees were generated from high quality single nucleotide variant (hqSNV) alignments by using SNVPhyl v1.0.1 to assess isolate relatedness (*5*). We identified the best reference by using mash v2.0 by identifying the genome with the lowest average mash distance to the entire group (i.e., centroid) (*6*). Each identified cluster was run separately to calculate cluster hqSNV distances and core genome estimates (*7*,*8*).

Phylogenetic trees with publicly available genomes were created by calling core genes with Prokka v1.14.5 and Roary v3.5.9 (*9*). A maximum likelihood phylogeny was generated on the concatenated core gene alignment by using RaxML v8.2.12 with parameters -m GTRCAT -f a -x 123 -N 100 (*10*). For visualization purposes, *A. baumannii, A. nosocomialis,* and *A. pittii* genomes were down sampled to 100 isolates and non-ACBC isolates were removed. Trees were visualized with Interactive Tree of Life (iTOL) and rooted at the midpoint (*11*).

References

1. Bushnell B. BBTools: a suite of fast, multithreaded bioinformatics tools designed for analysis of DNA and RNA sequence data. Berkeley (CA): Joint Genome Institute; 2018.

2. Chen S, Zhou Y, Chen Y, Gu J. fastp: an ultra-fast all-in-one FASTQ preprocessor. Bioinformatics. 2018;34:i884–90. PubMed https://doi.org/10.1093/bioinformatics/bty560

3. Bankevich A, Nurk S, Antipov D, Gurevich AA, Dvorkin M, Kulikov AS, et al. SPAdes: a new genome assembly algorithm and its applications to single-cell sequencing. J Comput Biol. 2012;19:455–77. PubMed https://doi.org/10.1089/cmb.2012.0021

4. Pritchard G. Taxonomy in diagnostics for food security: soft-rotting enterobacterial plant pathogens, Anal. Methods: 12 [cited 2023 Aug 2]. https://pubs.rsc.org/en/content/articlelanding/2016/ay/c5ay02550h

5. Petkau A, Mabon P, Sieffert C, Knox NC, Cabral J, Iskander M, et al. SNVPhyl: a single nucleotide variant phylogenomics pipeline for microbial genomic epidemiology. Microb Genom. 2017;3:e000116. PubMed https://doi.org/10.1099/mgen.0.000116

6. Seemann T. Prokka: rapid prokaryotic genome annotation. Bioinformatics. 2014;30:2068–9. PubMed https://doi.org/10.1093/bioinformatics/btu153

7. Ondov BD, Treangen TJ, Melsted P, Mallonee AB, Bergman NH, Koren S, et al. Mash: fast genome and metagenome distance estimation using MinHash. Genome Biol. 2016;17:132. PubMed https://doi.org/10.1186/s13059-016-0997-x

8. Halpin AL, McDonald LC, Elkins CA. Framing bacterial genomics for public health (care). J Clin Microbiol. 2021;59:e0013521. PubMed https://doi.org/10.1128/JCM.00135-21

9. Page AJ, Cummins CA, Hunt M, Wong VK, Reuter S, Holden MT, et al. Roary: rapid large-scale prokaryote pan genome analysis. Bioinformatics. 2015;31:3691–3. PubMed https://doi.org/10.1093/bioinformatics/btv421

10. Stamatakis A. RAxML version 8: a tool for phylogenetic analysis and post-analysis of large phylogenies. Bioinformatics. 2014;30:1312–3. PubMed https://doi.org/10.1093/bioinformatics/btu033

11. Letunic I, Bork P. Interactive Tree Of Life (iTOL) v4: recent updates and new developments. Nucleic Acids Res. 2019;47(W1):W256–9. PubMed https://doi.org/10.1093/nar/gkz239

Appendix Table 1. Strains implicated in transfusion-transmitted sepsis identified during environmental sampling of healthcare facilities and blood establishments

Environmental sample location	State	Year	Whole genome sequencing cluster
Platelet agitator	Connecticut	2018	Cluster B, *Staphylococus saprophiticus*
Platelet agitator	Utah	2018	Cluster 2, *Acinetobacter seifertii*
Platelet agitator	Utah	2018	Cluster 1, Novel *Acinetobacter calcoaceticus-baumannii* complex spp.
Platelet agitator	California	2019	Cluster 2, *Acinetobacter seifertii*
Platelet agitator	California	2019	Cluster 1, Novel *Acinetobacter calcoaceticus-baumannii* complex spp.
Platelet agitator	California	2019	Cluster A, *Staphylococus saprophiticus*
Quality control laboratory cart	California	2019	Cluster 2, *Acinetobacter seifertii*
Quality control laboratory cart	California	2019	Cluster A, *Staphylococus saprophiticus*
Platelet agitator	California	2019	Cluster A, *Staphylococus saprophiticus*
Platelet agitator	California	2019	Cluster 2, *Acinetobacter seifertii*
Platelet agitator	California	2019	Cluster 2, *Acinetobacter seifertii*
Platelet agitator	California	2019	Cluster 1, Novel *Acinetobacter calcoaceticus-baumannii* complex spp.
Exterior of platelet component bag	California	2019	Cluster B, *Staphylococus saprophiticus*
Exterior of platelet component bag	California	2019	Cluster 2, *Acinetobacter seifertii*
Quality control laboratory cart	California	2019	Cluster 2, *Acinetobacter seifertii*
Platelet agitator	California	2019	Cluster A, *Staphylococus saprophiticus*
Platelet agitator	California	2019	Cluster 2, *Acinetobacter seifertii*
Platelet transporter	California	2019	Cluster A, *Staphylococus saprophiticus*
Platelet transporter	California	2019	Cluster 2, *Acinetobacter seifertii*
Platelet agitator	California	2019	Cluster 2, *Acinetobacter seifertii*
Platelet agitator	California	2019	Cluster A, *Staphylococus saprophiticus*
Lining of bag from transport box used to move samples	Connecticut	2019	Cluster 2, *Acinetobacter seifertii*
Lining of bag from transport box used to move samples	Connecticut	2019	Cluster B, *Staphylococcus saprophyticus*
Exterior of unopened platelet collection kit	Connecticut	2019	Cluster 1, *Acinetobacter seifertii*
Platelet Transport Box	Utah	2019	Cluster 1, Novel *Acinetobacter calcoaceticus-baumannii* complex spp.
Exterior surface platelet collection bag	Utah	2019	Cluster 1, Novel *Acinetobacter calcoaceticus-baumannii* complex spp.
Sterile docking of the inner contents of an apheresis platelet unit	North Carolina	2020	Cluster 1, *Leclercia adecarboxylata*
Sterile docking of the inner contents of an apheresis platelet unit	North Carolina	2020	Cluster 1, *Leclercia adecarboxylata*
Sterile docking the inner contents of an apheresis platelet unit	North Carolina	2020	Cluster A, *Staphylococcus saprophyticus*
Port area of an apheresis platelet unit	North Carolina	2020	Cluster 2, *Leclercia adecarboxylata*

Environmental sample location	State	Year	Whole genome sequencing cluster
Port area of an apheresis platelet unit	North Carolina	2020	Cluster 1, *Leclercia adecarboxylata*
Culture collected by sterile docking of contents of an apheresis platelet unit	North Carolina	2020	Cluster 1, *Acinetobacter seifertii*
Culture collected by wet swab from the port area of outside of apheresis container	North Carolina	2020	Cluster 1, *Acinetobacter nosocomialis*
Culture collected by wet swab from the port area of outside of apheresis container	North Carolina	2020	Cluster B, *Staphylococcus saprophyticus*

Appendix Table 2. Strains implicated in transfusion transmitted sepsis identified during environmental monitoring of a platelet collection kit manufacturing facility, October 2021—November 2022

Sample description	Sample culture and whole-genome sequencing results
Surface sample #1	*Staphylococcus saprophyticus*; Cluster B
Surface sample #2	*S. saprophyticus*; Cluster B
Surface sample #3	*S. saprophyticus*; Cluster B
Surface sample #4	*S. saprophyticus*; Cluster B
Room air sample	*S. saprophyticus*; Cluster B
Solution bioburden #1	*S. saprophyticus*; Cluster B
Solution bioburden #2	*S. saprophyticus*; Cluster B
Filling room	*Acinetobacter* spp. underwent multilocus sequencing typing but not available for whole-genome sequencing

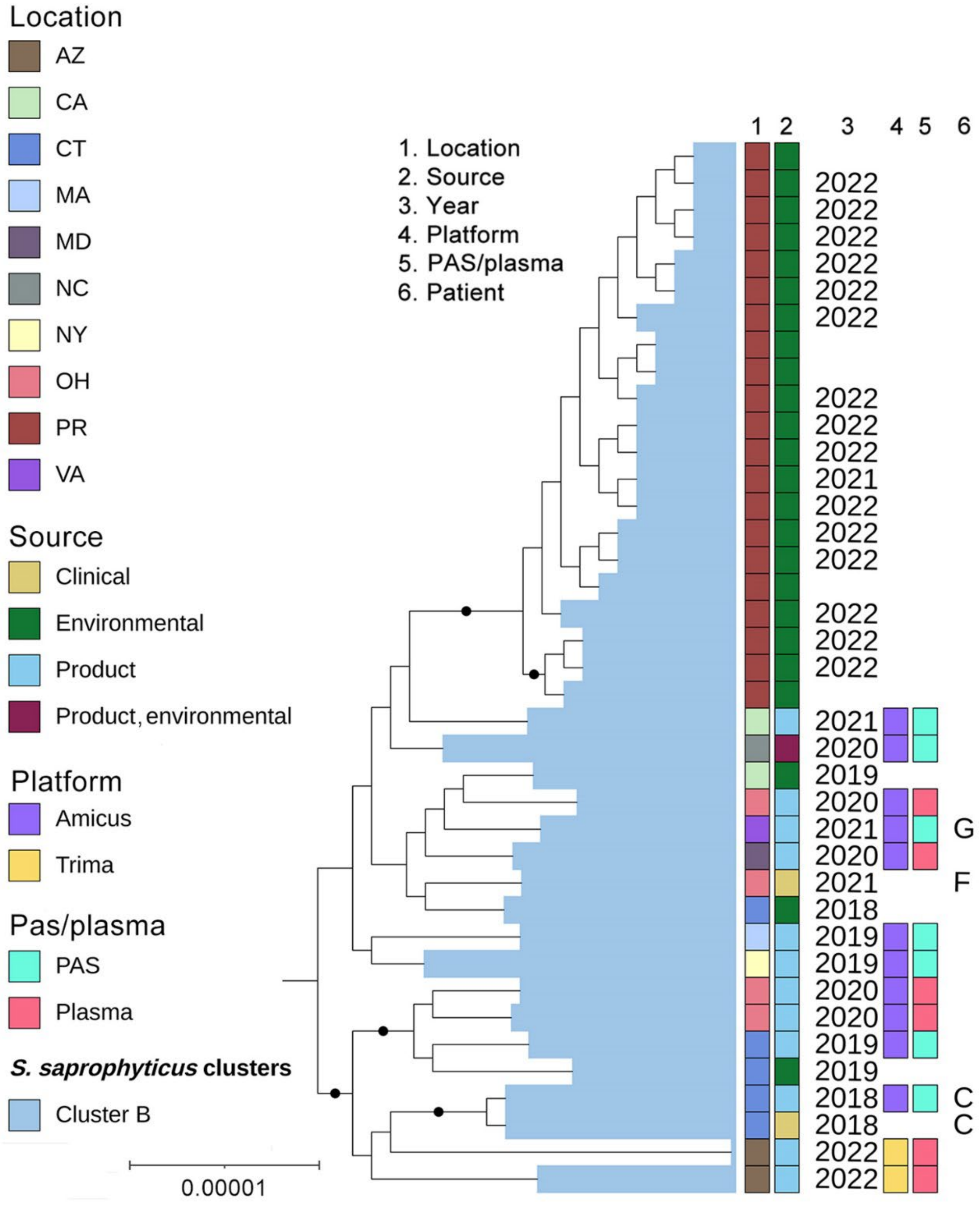

Appendix Figure. *Staphylococcus saprophyticus* cluster B core gene phylogeny. A RaxML–generated phylogeny based on 2,533 core genes from all *S. saprophyticus* cluster B genomes. Isolate location, isolate source, year, manufacturing platform, platelet additive solution (PAS) versus plasma for products were layered onto the phylogeny. Scale bar indicates nucleotide substitutions per site. Black circles on branches represent 100% support for the branch out of 100 bootstraps. PR, Puerto Rico. Other locations are state 2-letter codes.
